# Convergent and Divergent Functional Connectivity Patterns in Schizophrenia and Depression

**DOI:** 10.1371/journal.pone.0068250

**Published:** 2013-07-02

**Authors:** Yang Yu, Hui Shen, Ling-Li Zeng, Qiongmin Ma, Dewen Hu

**Affiliations:** College of Mechatronics and Automation, National University of Defense Technology, Changsha, Hunan, China; Hangzhou Normal University, China

## Abstract

Major depression and schizophrenia are two of the most serious psychiatric disorders and share similar behavioral symptoms. Whether these similar behavioral symptoms underlie any convergent psychiatric pathological mechanisms is not yet clear. To address this issue, this study sought to investigate the whole-brain resting-state functional magnetic resonance imaging (MRI) of major depression and schizophrenia by using multivariate pattern analysis. Thirty-two schizophrenic patients, 19 major depressive disorder patients and 38 healthy controls underwent resting-state functional MRI scanning. A support vector machine in conjunction with intrinsic discriminant analysis was used to solve the multi-classification problem, resulting in a correct classification rate of 80.9% via leave-one-out cross-validation. The depression and schizophrenia groups both showed altered functional connections associated with the medial prefrontal cortex, anterior cingulate cortex, thalamus, hippocampus, and cerebellum. However, the prefrontal cortex, amygdala, and temporal poles were found to be affected differently by major depression and schizophrenia. Our preliminary study suggests that altered connections within or across the default mode network and the cerebellum may account for the common behavioral symptoms between major depression and schizophrenia. In addition, connections associated with the prefrontal cortex and the affective network showed promise as biomarkers for discriminating between the two disorders.

## Introduction

Major depressive disorder (MDD) and schizophrenia are two of the most serious psychiatric disorders and share similar behavioral symptoms [1]. Some previous studies indicated that 59% of patients with schizophrenia met the DSM-III criteria for major or mild depression [2]. Similarly, other studies have suggested that patients with schizophrenia are 29 times more likely to have a lifetime diagnosis of MDD than the general population [3]. Symptoms and clinical findings, such as a depression factor [4], [5], [6], genetic risk [7], mild loss of brain volume [8], postnatal complications of brain development [9], lack of energy, anhedonia, and social withdrawal [10], often cause particular problems when attempting to differentiate between the two syndromes. Whether the similar behavioral symptoms underlie any convergent psychiatric pathological mechanisms is not yet clear, and few investigations have been performed to address this issue. Understanding the etiology and pathogenesis of schizophrenia and depression is a major challenge in the field of psychiatry [11]. Ciaran *et al* reported that the features of schizophrenia, especially those that are ‘negative’, exhibit many clinical similarities to the syndrome of depression [10]. Here, we speculated that schizophrenic and MDD patients share convergent dysfunctional connectivity patterns that account for their similar behavioral symptoms. Exploring the convergent and divergent functional connectivity patterns not only can enhance our comprehension of the pathophysiology of major depression and schizophrenia, but may also facilitate the clinical identification of major depression and schizophrenia, which is currently based largely on self-reported symptoms and clinical signs.

In recent years, resting-state functional magnetic resonance imaging (rs-fMRI) techniques have been widely used in the quantitative analysis of the brain in some neuropsychiatric disorders, including schizophrenia [12], [13] and MDD [14]. Hypotheses regarding functional connectivity abnormalities have been proposed as physiological explanations of the behavioral syndromes of MDD patients [15], [16]. Furthermore, rs-fMRI studies have detected resting-state network (RSN) alterations, specifically, abnormalities in the default mode network (DMN) [17], [18], affective network [19], and visual cortical areas [14] in MDD patients. Similarly, hypotheses regarding functional connectivity abnormalities in schizophrenic patients have been investigated in many other neuroimaging studies [12], [20]. For example, Whitfield-Gabrieli *et al* identified abnormal connectivity within the DMN in schizophrenic patients compared with healthy controls [20], and Salvador *et al* found that some regions of the DMN showed hyper-connectivity in schizophrenic patients [21]. These studies accelerated the search for pathophysiological mechanisms of MDD or schizophrenia and supplied some additional information for current clinical diagnostic systems which are mainly based on the patients’ clinical manifestations [14], [22]. From the above-mentioned studies, we noticed that MDD and schizophrenia exhibited convergent abnormal connections associated with the same regions, such as the prefrontal lobe [17], [23], [24], thalamus [16], [25], and hippocampus [26]. However, whether the two disorders share convergent, in addition to divergent, functional connectivity patterns has not been well investigated. The present study sought to investigate the whole-brain rs-fMRI of major depression and schizophrenia using multivariate pattern analysis.

Multivariate pattern analysis has generated great interest due to its capacity to identify potential neuroimaging-based biomarkers to differentiate patients from healthy controls at the individual subject level, as well as its ability to detect spatially distributed information to further highlight the neural mechanisms that underlie patients’ behavioral symptoms [27], [28], [29], [30], which can complete previous group-level statistical analysis studies. Recent studies have used multivariate pattern analysis to explore structural and functional alterations in schizophrenia or MDD, obtaining satisfactory correct classification rates [14], [31]. In the current study, multivariate pattern analysis was further extended to the multiclass discriminative analysis of whole-brain resting-state functional connectivity in schizophrenic patients, MDD patients, and healthy controls to explore the convergent and divergent functional connectivity patterns of schizophrenia and MDD. Machine learning is an important aspect of multivariate pattern analysis. In the last few years, several learning techniques have been widely used in the multivariate pattern analysis of rs-fMRI, such as principal component analysis (PCA) [32], independent component analysis (ICA) [33], and the multivariate linear model [22]. However, these above-mentioned methods do not analyze the differences in functional connections, which are more significant in real-word discriminative analysis. In the current study, we first used intrinsic discriminative analysis (IDA) [34] in the multivariate pattern analysis of rs-fMRI data. IDA is a supervised linear dimensionality reduction method that explicitly exploits the knowledge of which types of features are critical for classification and tries to best differentiate different classes by maximizing the inter-class difference while minimizing the intra-class difference.

Due to the limited samples size, the leave-one-out cross-validation (Loocv) strategy was used during the dimensionality reduction process. When the dataset of features in the embedding space was obtained, support vector machines (SVMs) with a linear kernel function were employed to solve the classification problems [35]. Functional connectivity features with the highest discriminative power were further identified using a reconstruction technique.

## Materials and Methods

### Participants

This study was approved by the Ethics Committee of the Second Xiangya Hospital of Central South University, with knowing that the participants, including schizophrenic and MDD patients, belonged to a traditionally vulnerable population. Participants, including 19 MDD patients, 32 schizophrenic patients, and 38 healthy controls, were physically healthy as indicated by physical examinations performed before screening. All subjects provided their written informed consent after receiving a complete description of this study. The patients provided informed consent with accompany of their next of kin, and their capacity of providing informed consent was confirmed by clinical psychiatrists. Because the patients belong to a traditionally vulnerable population, their next of kin also provided informed consent to participant in this study on behalf of them. MDD and schizophrenic patients were recruited from outpatient departments and inpatient units at the Second Xiangya Hospital of Central South University. All of the patients fulfilled the criteria for schizophrenia or MDD according to the DSM-IV (Diagnostic and Statistical Manual of Mental Disorders, Fourth Edition) [36], and confirmation of the diagnosis was made by clinical psychiatrists. Symptom severity for the schizophrenic patients was assessed with the Positive and Negative Syndrome Scale (PANSS) [37]. The depressive symptoms of the MDD patients were assessed with the 17-item Hamilton Depression Rating Scale (HDRS) on the days of the scans [38]. Exclusion criteria included acute physical illness, substance abuse or dependence, a history of head injury resulting in loss of consciousness, and a major psychiatric or neurological illness other than schizophrenia or depression. Similar exclusion criteria were adopted for healthy control subjects, who were recruited via advertisement and matched to the affected individuals with respect to age, gender, education level, and handedness (details are shown in Table 1).

**Table 1:** Demographic and clinical profiles of the participants in this study.

variable	MDD patients	Schizophrenia	Healthy controls
Gender (M/F)	11/8	25/7	27/11
Age (years)	26.65(±7.62)	24±5.66	24.44±4.45
Education (years)	12.41(±2.24)	11.15±2.50	13.65±2.78
PANSS score	––	80.06±16.55	––
HRSD score	25.43 (±6.34)	––	––

PANSS: Positive and Negative Syndrome Scale; HDRS: Hamilton Depression Rating Scale.

### Resting Experiment and Data Acquisition

fMRI scans were performed with a 1.5-T GE Signa System (GE Signa, Milwaukee, Wisconsin, USA) using a gradient-echo echo planar imaging sequence. The imaging parameters were as follows: TR = 2000 ms, TE = 40 ms, FOV = 24 cm, FA = 90°, matrix = 64×64, slice thickness = 5 mm, gap = 1 mm, slices = 20. During the experiment, the subjects were instructed to relax, keep their eyes closed, remain awake, and perform no specific cognitive exercises. Foam pads and earplugs were used to minimize head motion and scanner noise, respectively. Each functional resting-state session lasted ∼6 minutes, resulting in 180 volumes.

### Data Preprocessing

Image preprocessing was performed using the statistical parametric mapping software package (SPM8, Welcome Department of Cognitive Neurology, Institute of Neurology, London, UK, http://www.fil.ion.ucl.ac.uk/spm). For each subject, the first five volumes of the scanned data were discarded due to magnetic saturation effects. The remaining volumes were corrected by registering and reslicing them to account for head movement. Then, the volumes were normalized to the standard echo planar imaging template in the Montreal Neurological Institute space. The resulting images were spatially smoothed with a Gaussian filter of an 8-mm full-width half-maximum kernel and then temporally filtered with a Chebyshev band-pass filter (0.01–0.08 Hz). The registered fMRI volumes were further divided into 116 regions according to the anatomically labeled atlas previously validated and reported by Tzourio-Mazoyer *et al* [39]. The atlas divides the cerebrum into 90 regions (45 in each hemisphere) and divides the cerebellum into 26 regions (9 in each cerebellar hemisphere and 8 in the vermis).

### Functional Connectivity

Regional mean time series were acquired for each individual by averaging the fMRI time series over all voxels within each of the 116 regions. For each regional mean time series, we performed further regression of the global mean signals and assessed the effects of translations and rotations of the head as estimated in the course of initial movement correction by image realignment. The residuals of the above regressions constituted the set of regional mean time series used for further functional connectivity analysis [40]. We then evaluated the functional connection between each pair of regions using Pearson’s correlation coefficient, resulting in a 6670-dimensional feature vector for each subject.

### IDA Algorithm and Intrinsicconnectomes

Suppose that there are 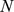 training samples sampled from 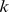 classes (each class has 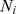, (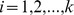) samples and 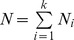) in the original high-dimensional space and that 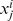 in 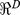 denotes the *j*th sample in the *i*th class.

We now consider the following matrixes:
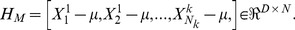
(1)

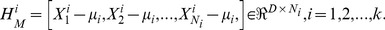
(2)where



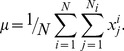
(3)

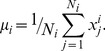
(4)


Suppose that 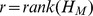 and that the singular value decomposition of 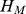 is 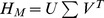, where *U* and 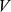 are orthogonal. Partitioning 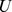, 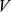, and 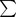 results in the following:


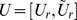
, where 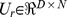 and 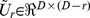.


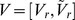
, where 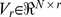 and 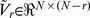.


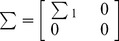
, where 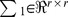 is nonsingular.

Similarly, we suppose that 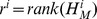 and acquire 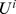 from the SVD of matrix 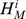 (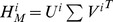).

After performing the above calculation, we can decompose 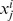 into the following form:
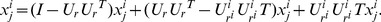
(5)where 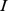 is the identity matrix.

This formula describes the three components of the original sample after being decomposed. It has been proven that these three components correspond to different properties. 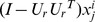 is a common component corresponding to the common nature of all the training samples, 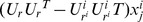 is a common component of all the training samples belonging to the same class, and 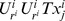 can be viewed as the difference of an individual in a particular class. Thus, the intrinsic model is established.

Eq. (5) can then be rewritten as the following formula when projecting the sample onto a lower d-dimensional space by using a 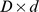 transformation matrix 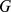.
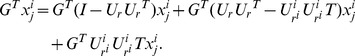
(6)


The transformation matrix 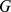 can be obtained by solving the maximization problem of the following objective function:
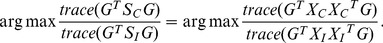
(7)where



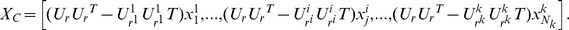
and



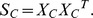


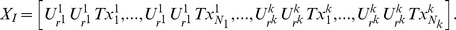
and



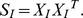



The objective function is usually approximated as the following form because Eq. (7) does not often have a closed-form solution.
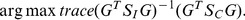
(8)


The dimensionality reduction algorithm based on the above intrinsic discriminant criterion is called intrinsic discriminant analysis.

The optimal transformation vectors 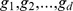 are given by solving the following generalized eigenvector problem:
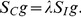
(9)


However, 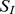 is argued to be singular when the number of training samples is smaller than the dimensionality. The perturbation technique was introduced to overcome this problem, that is, we changed Eq. (9) to the following form:
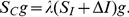
(10)where 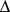 is a relatively small positive number and 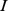 is the identity matrix.

Obtaining the transportation vectors 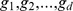 corresponding to the 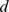 maximum eigenvalue solutions, the final transformation matrix 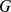 from the original sample space to the embedded space is formed as:
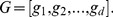
(11)


The IDA algorithm procedure is formally stated as:

Step 1. Without loss of generality, we store the training samples of a certain class compactly in the original training data matrix 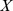 using order rearrangement, i.e., 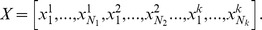


Step 2. The SVD of 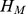 and 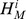 (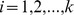) is computed to obtain 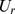 and 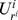. Then, 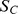 and 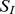 are computed using Eq. (7).

Step 3. The generalized eigenvector problem is solved, and the transportation matrix 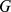 is obtained.

The column vectors of 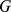 are called intrinsicconnectomes in subsequent discussion.

### Multiclass Classification Based on Intrinsic Discriminative Analysis

**Figure 1:**
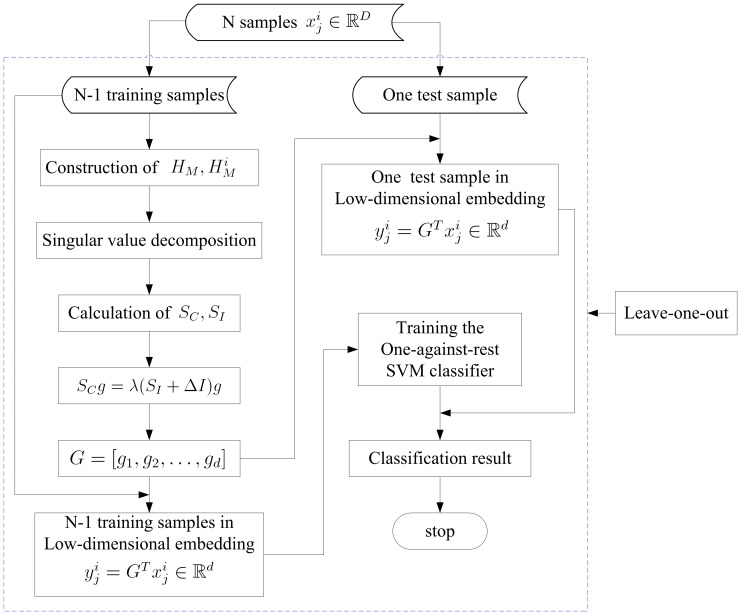
Flow chart of the intrinsicconnectome method.

The multivariate pattern analysis of fMRI is a challenging task due to the high dimensionality of the data. IDA was applied here to reduce the dimensionality of the original feature space. When the data set of features in the embedding coordinate were obtained, SVM with a linear kernel function was employed to solve the classification problem [35]. A one-against-rest strategy was used to design our classifiers [41]. For a k-class problem, the one-against-rest method constructs k SVM models. The *i*th SVM is trained with the training samples in the *i*th class with positive labels and other samples with negative labels. The final output of the one-against-rest method is the class that corresponds to the SVM with the highest output value. A Loocv strategy was employed to estimate the generalization ability of the classifiers. A flowchart of our method is shown in Fig. 1. To comprehensively investigate the discriminative ability of the IDA method, the classification performance of our IDA algorithm was compared with that of the PCA method.

### Identification of Features with High Discriminative Power

We determined the functional connection features with the highest discriminative power via the reconstruction technique based on the performance of each one-against-rest classifier. Because each feature influences the classification by its weight, the larger the absolute magnitude of a feature’s weight, the more it will affect the classification result. For each one-against-rest classifier, we obtained a weight vector in each Loocv experiment. The weight vector for this one-against-rest classifier was finally acquired by averaging the aforementioned weight vectors. We therefore obtained three weight vectors representing the features’ discriminative power for each one-against-rest classifier. Because we performed the classification in the dimension-reduced subspace, to determine the original functional connections that make significant contributions to the classification, we then mapped back each weight vector to the original high-dimensional space. Thus, for all of the 6670 resting-state functional connections, we obtained the order of their contribution to the classification for each one-against-rest classifier. In the subsequent study, the top 5% of functional connection features with the greatest weights were extracted as the most discriminative functional connections. Region weight, representing the relative contribution of each region to the classification, was denoted by its occurrence number in the consensus functional connections.

## Results

### Intrinsicconnectomes

**Figure 2:**
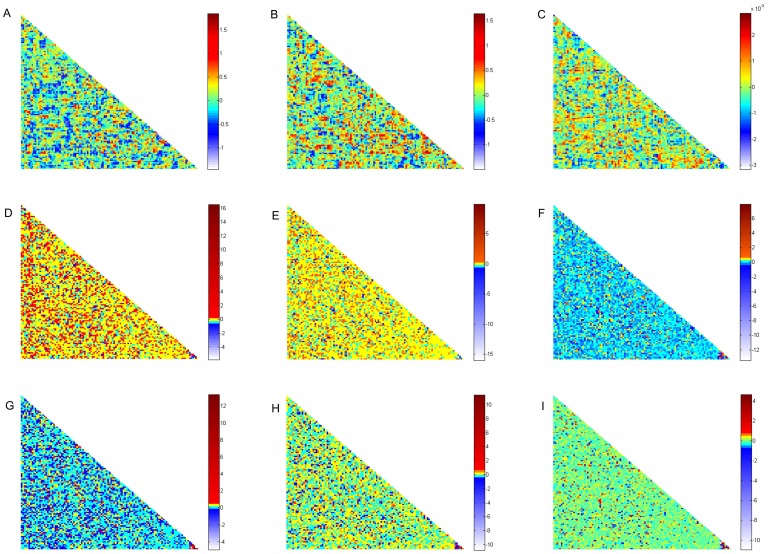
The first nine intrinsicconnectomes calculated by our method.

According to theoretical analysis, the columns of the transformation matrix 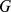 are called intrinsicconnectomes, and any functional connections can be represented as a combination of these intrinsicconnectomes. We displayed the first nine intrinsicconnectomes in Fig. 2.

### Classification Results

**Figure 3:**
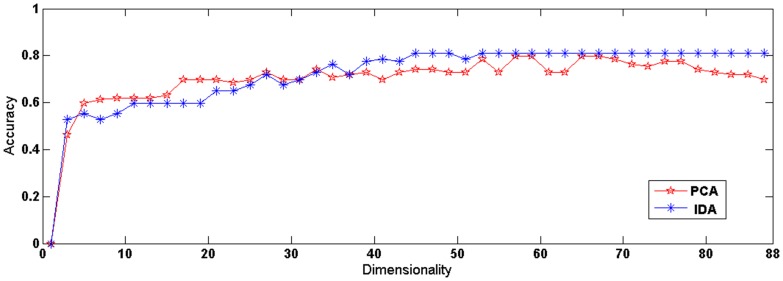
The curve of classification accuracy of the IDA and PCA methods.

As we described in the Materials and methods section, a total of 89 samples were used in the discriminative analysis in this work, and by using Loocv, our IDA method achieved an accuracy of 80.9% at the individual level (84.2% for MDD patients, 78.9% for healthy controls, and 81.3% for schizophrenic patients; details are shown in Table 2). The dimensionality of the embedding space was set using a grid search format [2, 88] with a granularity of 1. All of the results were reported based on the best parameter settings. To further validate the discriminative ability of the IDA method, we compared the IDA algorithm with the traditional PCA method, which achieved an accuracy of 79.8%. The best classification rates for the two methods, along with their associated dimensionality, are summarized in Fig. 3.

**Table 2:** Confusion matrix for results in leave-one-out cross-validation.

Classes	MDD	Healthy controls	Schizophrenia
MDD	84.2%	5.3%	10.5%
Healthy controls	13.2%	78.9%	7.9%
Schizophrenia	12.5%	6.2%	81.3%

The rows of this matrix indicate the groups of the subjects (ground truth), and the columns indicate the predictions by the classifier. The cells in each row contain the proportion of trials in which subjects responded with the category indicated by the column.

### Functional Connections with High Discriminative Power

**Figure 4:**
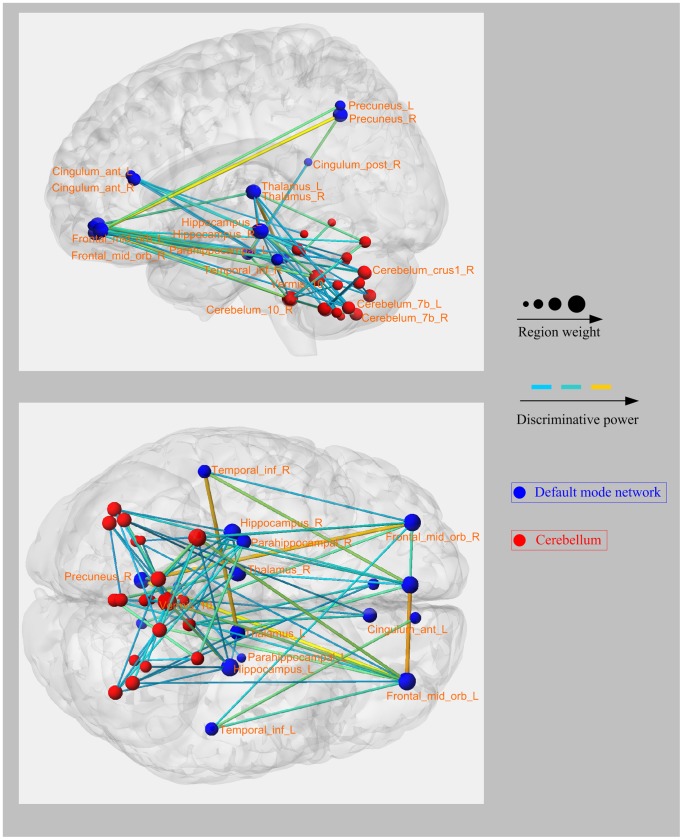
Left and bottom views of the convergent functional connectivity patterns with high discriminative power. Regions are color-coded by category. The line colors representing the discriminative power of the relative connections are scaled with their mean discriminative power in the leave-one-out cross-validation.

**Figure 5:**
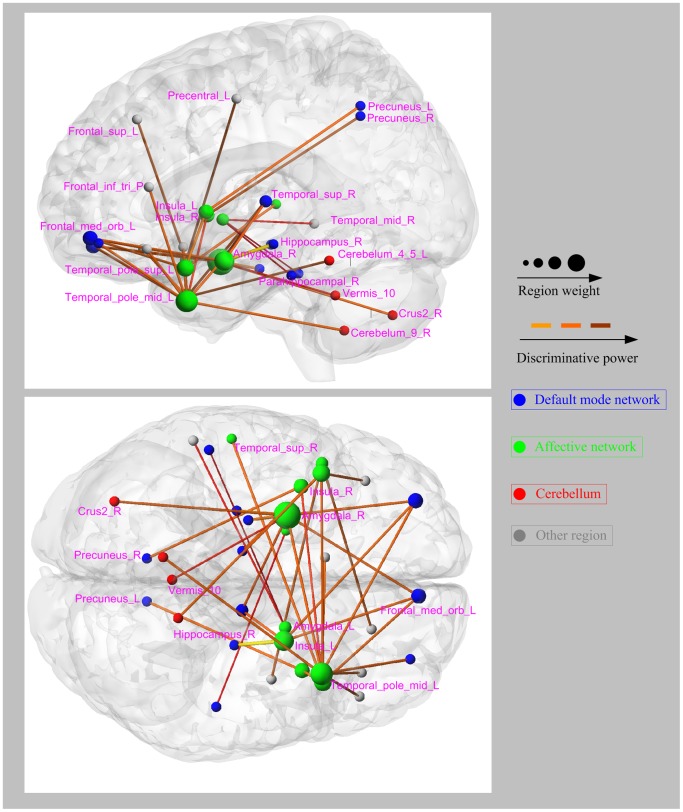
Left and bottom views of the divergent functional connectivity patterns with high discriminative power. Regions are color-coded by category. The line colors representing the discriminative power of the relative connections are scaled with their mean discriminative power in the leave-one-out cross-validation.

In this study, 5% consensus functional connections were identified for each one-against-rest classifier. Compared with the healthy controls, the MDD and schizophrenia patients showed convergent altered functional connectivity patterns related to the DMN (mainly containing the parahippocampal gyrus, precuneus, anterior cingulate cortex, hippocampus, thalamus, inferior temporal gyrus, posterior cingulate cortex, and medial prefrontal cortex), as well as the cerebellum. Several brain regions exhibited greater weights than others (i.e., the medial prefrontal cortex, anterior cingulate cortex, thalamus, hippocampus, cerebelum_7b, cerebelum_9, cerebelum_vermis10, and cerebelum_crus1). Those functional connections with high discriminative power were observed within or across the DMN and the cerebellum (Fig. 4), i.e., connections between the medial prefrontal cortex and the precuneus, thalamus, and inferior temporal gyrus; connections between the thalamus and the anterior cingulate cortex and hippocampus; as well as connections between the cerebellum and the medial prefrontal cortex, hippocampus, thalamus, and anterior cingulate cortex. Divergent functional connectivity patterns that make key contributions to the identification of schizophrenic and MDD patients were obtained by combining the MDD-against-rest classifier and the schizophrenia-against-rest classifier. Some consensus functional connections related to the prefrontal cortex, temporal poles, and amygdala were identified. Such connections included connections between the amygdala and the medial prefrontal cortex, parahippocampus, hippocampus, precuneus, and some cerebellar regions, as well as connections between the temporal poles, amygdala, and medial prefrontal cortex (Fig. 5).

## Discussion

The rs-fMRI technique is currently generated increasing interest because the imaging of baseline states is fundamental in understanding human brain functions [42]. In the present study, rs-fMRI was extended to the field of discriminative analysis of schizophrenia and depression to identify the potential convergent and divergent functional connectivity patterns associated with the two disorders. Selecting low-dimensional embedding of rs-fMRI acquired using IDA as classification features, we have designed a data-driven multiclass classifier and successfully extracted the significant discriminative functional connections that potentially underlie the spontaneous neural activities in the brains of MDD patients, schizophrenic patients, and healthy controls. Our preliminary study suggested that resting-state functional connections are promising for use in classifying schizophrenic patients, MDD patients, and healthy controls, and, accordingly, rs-fMRI could be potentially useful in revealing the convergent and divergent functional connectivity patterns associated with the two disorders.

In recent years, the multivariate pattern analysis method has been widely used in neuroimaging studies [28], [30]. Compared with the group comparison methods, which have been previously used in some studies to explore abnormal function connectivity to differentiate patients from healthy controls, there are many benefits of using multivariate pattern analysis. First, group-level statistical methods are less helpful in diagnosis due to complex dysfunction in the entire brain in psychiatric disorders [14], [25], [31], [43]. Second, group-level methods are not helpful in clinical diagnosis at the individual subject level. In recent years, multivariate pattern analysis methods have been widely used in neuroimaging studies, as they can not only identify potential neuroimaging-based biomarkers with which to differentiate patients from healthy controls at the individual subject level, but they can also detect interesting spatially distributed information to further highlight the neural mechanisms underlying the behavioral symptoms of MDD and schizophrenia. Our previous studies have used multivariate pattern analysis to explore the resting-state functional alterations in schizophrenia and depression compared with the general population, obtaining satisfactory correct classification rates [14], [31]. In the present study, we extended the multivariate pattern analysis method to the multiclass discriminative analysis of rs-fMRI to explore the convergent and divergent functional connectivity patterns in schizophrenic and MDD patients. To our knowledge, this study was the first to use the IDA algorithm in the discriminative analysis of rs-fMRI data, which may provide some useful insights into how to address the challenging problem of the dimensionality reduction of rs-fMRI data. IDA is a supervised linear dimensionality reduction. Compared with nonlinear algorithms, linear algorithms were suggested to be more insensitive to over-fitting problems, especially in case with a large number of dimensions and small sample sizes [44]. Another advantage of linear algorithms is that they can reveal potential neuroimaging-based biomarkers using the reconstruction technique. On the basis of their experimental results, Young Wang *et al*. suggested that IDA exhibited better performance than PCA [34]. Our experimental results also showed a slight superiority of IDA over the PCA method. The most likely explanation for the superiority of IDA in performance compared with the unsupervised PCA algorithm is that IDA explicitly uses data label information to supervise the implementation of dimensionality reduction. In other words, IDA uses the knowledge of which types of functional connections are critical for discriminative analysis, which in theory can lead to better discriminative performance. In this aspect, IDA is more generalized and suitable in multi-class discriminative analysis. However, the difference between the two methods is, in practice, only one sample. Accordingly, it would be very important to evaluate the performance of the IDA method with a larger sample size using neuroimaging data in the future.

A number of previous studies have identified abnormalities in the function of the DMN in schizophrenia and MDD, such as abnormalities in the bilateral hippocampus [18], anterior cingulate cortex, thalamus, inferior temporal gyrus, and posterior cingulate cortex [14], [16] in MDD patients, as well as abnormalities in the hippocampus [45], medial prefrontal cortex, anterior cingulate cortex [24], thalamus, and posterior cingulate cortex [46] in schizophrenic patients. Regarding the convergent functional connectivity patterns, regions with high discriminative power related to the DMN were consistent with these previous findings. The DMN is known to be involved in self-referential activity [47], [48]. Furthermore, ample evidence suggests that the anterior cingulate cortex, a key component of the DMN, plays a distinctive role in cognitive functions and information processing [49], [50]. Altered functional connections related to the anterior cingulate cortex have been implicated as a focus of dysfunction in MDD [16], [18] and schizophrenic [23] patients. The hippocampus has been suggested to be critical in memory formation [51], and it is involved in the deficits in working memory observed in MDD [52] and schizophrenic [53] patients. Although schizophrenia and depression are conventionally viewed as two distinct disorders, similar symptoms are evident during the disease courses [54]. Ciaran *et al* suggested that depression and schizophrenia are not simply two independent illnesses, and moreover, that the neurobiology of depressive symptoms in schizophrenia may have similarities to that of depressive disorder [10]. Kohler *et al* suggested that frontal lobe dysfunction may account for the depressive symptom in schizophrenia [55]. Previous studies have also provided presumptive evidence for the common pathology of schizophrenia and MDD, particularly in the prefrontal cortex, for example, a reduction in grey matter volume, changes in glucose metabolism, and changes in blood flow have been reported in the prefrontal cortex in both schizophrenia [56], [57] and MDD [58]. Accordingly, we assumed that convergent dysfunctions of the identified brain regions may reveal the convergent neuroimaging-based pathological mechanisms of depression and schizophrenia. Abnormalities in the cerebellum have been previously reported in schizophrenic and MDD patients [19], [59], [60]. A previous study also suggested that the cerebellum is involved in cognitive and emotional activities [61]. In our study, compared with the healthy controls, the connections between the cerebellum (cerebelum_7b, cerebelum_9, cerebelum_vermis10, and cerebelum_crus1) and some cerebral regions exhibited very high discriminative power in both the MDD and schizophrenic patients. Therefore, we speculated that the convergent aberrant connections between the cerebellum and the cerebral cortex may contribute to part of the common behavioral symptoms in MDD and schizophrenia. Further work is required to clarify our speculation.

Resting-state functional connections between the amygdala and prefrontal cortex, hippocampus, and precuneus, as well as connections between the temporal poles and the amygdala and the prefrontal cortex, were found to be different between the MDD and schizophrenic patients. A large number of studies have reported that people with schizophrenia demonstrate dysfunction of the prefrontal cortex during the performance of working memory tasks [62], [63]. Although other reports have suggested that people with major depression also display prefrontal cortex deficits [64], these deficits were reported to be not as severe as those found in schizophrenia [65]. A recent fMRI study suggested that the deficits in prefrontal cortex function are different in schizophrenia and MDD [66]. Consistent with this study, we predicted that schizophrenic and MDD patients exhibit different functional impairments in the prefrontal cortex. The affective network is known to be involved in mood regulation and affective processing [67]. The amygdala has a wide variety of functions, such as cognition, memory consolidation, and control of affective behaviors [68], [69]. Morphometric and histological abnormalities have been reported in the amygdala of patients with schizophrenia [70]. Meta-analyses also showed volume reductions in the amygdala in patients with schizophrenia [70]. Other studies argued that abnormal connections related to the amygdala may affect the regulation of mood [71] and reflect dysfunctions in visceral monitoring, which is compromised in MDD [18]. However, our experimental results suggested that the amygdala was affected differently in schizophrenia and depression. Abnormalities in the amygdala in patients with these psychiatric diseases may be pathophysiologically explained as follows. On one hand, abnormalities of the amygdala constitute a universal phenomenon throughout the brain of psychiatric patients, which may account for the respective physiological mechanisms of MDD and schizophrenia. On the other hand, the functional integration of the amygdala is differently affected between the two disorders. A recent study revealed that fatty acid metabolism in the amygdala is different between MDD and schizophrenia [72], which partially substantiates our explanation. Moreover, with respect to the temporal poles, some studies suggested that their dysfunctions are associated with clinical symptoms in schizophrenia [73]. Both the amygdala and the temporal pole are coincidently located within the affective network (including the amygdala, temporal poles, pallidum, insula, and superior temporal gyrus). Accordingly, we speculated that the functional integration of the prefrontal cortex and the affective network may be differently affected in the MDD and schizophrenic patients. The difference may partially reflect the different emotional and cognitive behavioral symptoms seen between MDD and schizophrenic patients and may enable the development of neuroimaging-based approaches to distinguish the two patient groups. This preliminary speculation needs to be further investigated.

### Limitations

Although the classification results of the present study are encouraging, several possible limitations still need to be considered. First, due to the limited sample size, it is important to test the classification performance of our methods and to confirm our findings with a larger sample size in the future. Second, global signal regression was applied in the data preprocessing for the consideration of removing potential sources of physiological noise. However, the success of this technique has been variable between studies. For example, Fox *et al* used global signal regression in functional connectivity analysis and suggested that this technique may remove several potential sources of physiological noise [74]. Therefore, there is much work being conducted in the use of global signal regression to correct for physiological confounders. Murphy *et al* suggested that this technique may result in a negative mean correlation value in functional connectivity analysis such that it may consequently change the inherent functional connectivity patterns [75]. Based on their experimental results, Saad *et al* suggested that this technique can induce false anti-correlations and even induce false between-group differences. Therefore, we performed the classification without removing the global signal. Keeping the parameters unchanged, the results showed that the overall classification accuracy did not differ from the previous accuracies.

### Conclusions

In the present study, we designed a data-driven multiclass classifier based on the intrinsicconnectomes model and successfully extracted the significant discriminative functional connections that potentially underlie spontaneous neural activity in the brains of MDD and schizophrenic patients, which should shed new light on how to address the challenging problem of dimensionality reduction of rs-fMRI data. Moreover, this preliminary study suggested that rs-fMRI could be potentially useful in revealing the convergent and divergent functional connectivity patterns of schizophrenia and depression. Compared with the healthy controls, the MDD and schizophrenic patients both exhibited altered functional connections within or across the DMN and the cerebellum, which may account for the similar behavioral symptoms seen in the two disorders. In addition, our results demonstrated that the prefrontal cortex and the affective network played notable roles in distinguishing the MDD patients from the schizophrenic patients. Further work is required to clarify these findings.

## References

[1] Häfner H, Maurer K, Trendler G, Heiden Wad, Schmidt M (2005) The early course of schizophrenia and depression. Eur Arch Psychiatry Clin Neurosci 255: 167–173. 15995900 10.1007/s00406-005-0584-8

[2] Kessler RC, McGonagle KA, Zhao S, Nelson CB, Hughes M, et al (1994) Lifetime and 12-month prevalence of DSM-III-R psychiatric disorders in the United States. Results from the National Comorbidity Survey. Psychiatry 51: 8–19. 10.1001/archpsyc.1994.03950010008002 8279933

[3] Robins LN, Regier DA (1991) Psychiatric Disorders in America: The Epidemiological Catchment Area Study. New York: Free Press.

[4] Van OJ, Verdoux H, Maurice-Tison S, Gay B, Liraud F, et al (1999) Self-reported psychosis-like symptoms and the continuum of psychosis. Soc Psychiatry Psychiatr Epidemiol 34: 459–463. 10541665 10.1007/s001270050220

[5] Liddle PF (1987) Schizophrenic syndromes, cognitive performance and neurological dysfunction. Psychol Med 17: 49–57. 3575577 10.1017/s0033291700012976

[6] Liddle PF (1987) The symptoms of chronic schizophrenia: a re-examination of the positive-negative dichotomy. Br J Psychiatry 151: 145–151. 3690102 10.1192/bjp.151.2.145

[7] Maier W, Lichtermann D, Franke P, Heun R, Falkai P, et al (2002) The dichotomy of schizophrenia and affective disorders in extended pedigrees. Schizophr Res 57: 259–266. 12223257 10.1016/s0920-9964(01)00288-2

[8] Heckers S, Stone D, Walsh J, Shick J, Koul P, et al (2002) Differential hippocampal expression of glutamic acid decarboxylase 65 and 67 messenger RNA in bipolar disorder and schizophrenia. Arch Gen Psychiatry 59: 521–529. 12044194 10.1001/archpsyc.59.6.521

[9] Elkis H, Friedman L, Wise A, Meltzer H (1995) Meta-analysis of studies of ventricular enlargement and cortical sulcal prominence in mood disorders Comparisons with controls or patients with schizophrenia. Arch Gen Psychiatry 52: 735–746. 7654125 10.1001/archpsyc.1995.03950210029008

[10] Mulholland C, Cooper S (2000) The symptom of depression in schizophrenia and its management. Adv Psychiatr Treat 6: 169–177.

[11] Angelucci F, Brené S, Mathé A (2005) BDNF in schizophrenia, depression and corresponding animal models. Molecular psychiatry 10: 345–352. 15655562 10.1038/sj.mp.4001637

[12] Woodward ND, Rogers B, Heckers S (2011) Functional resting-state networks are differentially affected in schizophrenia. Schizophr Res 130: 86–93. 21458238 10.1016/j.schres.2011.03.010 PMC3139756

[13] Meda S, Stevens M, Folley B, Calhoun V, Pearlson G (2009) Evidence for anomalous network connectivity during working memory encoding in schizophrenia: An ICA based analysis. Plos One 4: e7911. 19936244 10.1371/journal.pone.0007911 PMC2775682

[14] Zeng L-L, Shen H, Liu L, Wang L, Li B, et al (2012) Identifying major depression using whole-brain functional connectivity: a multivariate pattern analysis. Brain 135: 1498–1507. 22418737 10.1093/brain/aws059

[15] Anand A, Li Y, Wang Y, Gao S, Wu JW, et al (2005) Genetic correlates of cortico-limbic activity and connectivity in major depression. Neuropsychopharmacology 30: S159–S159.

[16] Greicius MD, Flores BH, Menon V, Glover GH, Solvason HB, et al (2007) Resting-State Functional Connectivity in Major Depression: Abnormally Increased Contributions from Subgenual Cingulate Cortex and Thalamus. Biol Psychiatry 62: 429–437. 17210143 10.1016/j.biopsych.2006.09.020 PMC2001244

[17] Bluhm R, Williamson P, Lanius R, Theberge J, Densmore M, et al (2009) Resting state default-mode network connectivity in early depression using a seed region-of-interest analysis: decreased connectivity with caudate nucleus. Psychiatry Clin Neurosci 63: 754–761. 20021629 10.1111/j.1440-1819.2009.02030.x

[18] Sheline YI, Price JL, Yan ZZ, Mintun MA (2010) Resting-state functional MRI in depression unmasks increased connectivity between networks via the dorsal nexus. Proc Natl Acad Sci USA 107: 11020–11025. 20534464 10.1073/pnas.1000446107 PMC2890754

[19] Mayberg HS (2003) Modulating dysfunctional limbic-cortical circuits in depression: towards development of brain-based algorithms for diagnosis and optimised treatment. Br Med Bull 65: 193–207. 12697626 10.1093/bmb/65.1.193

[20] Whitfield-Gabrieli S, Thermenos HW, Milanovic S, Tsuang MT, Faraone SV, et al (2009) Hyperactivity and hyperconnectivity of the default network in schizophrenia and in first-degree relatives of persons with schizophrenia. Proc Natl Acad Sci USA 106(4): 1279–1284. 19164577 10.1073/pnas.0809141106 PMC2633557

[21] Salvador R, Sarro S, Gomar JJ, Ortiz-Gil J, Vila F, et al (2010) Overall brain connectivity maps show cortico-subcortical abnormalities in schizophrenia. Hum Brain Mapp 31: 2003–2014. 20225222 10.1002/hbm.20993 PMC6870792

[22] Kawasaki Y, Suzuki M, Kherif F, Takahashi T (2007) Multivariate voxel-based morphometry successfully differentiates schizophrenia patients from healthy controls. NeuroImage 34: 235–242. 17045492 10.1016/j.neuroimage.2006.08.018

[23] Boksman K, Théberge J, Williamson P, Drost DJ, Malla A, et al (2005) A 4.0-T fMRI study of brain connectivity during word fluency in first-episode schizophrenia. Schizophr Res 75: 247–263. 15885517 10.1016/j.schres.2004.09.025

[24] Garrity AG, Pearlson GD, McKiernan K, Lloyd D, Kiehl KA, et al (2007) Aberrant “default mode” functional connectivity in schizophrenia. Am J Psychiatry 164: 450–475. 17329470 10.1176/ajp.2007.164.3.450

[25] Bluhm RL, Miller J, Lanius RA, Osuch EA, Boksman K, et al (2007) Spontaneous low-frequency fluctuations in the BOLD signal in schizophrenic patients: Anomalies in the default network. Schizophr Bull 33(4): 1004–1012. 17556752 10.1093/schbul/sbm052 PMC2632312

[26] Micheloyannis S, Pachou E, Stam CJ, Breakspear M, Bitsios P, et al (2006) Small-world networks and disturbed functional connectivity in schizophrenia. Schizophr Res 87: 60–66. 16875801 10.1016/j.schres.2006.06.028

[27] Fan Y, Shen DG, Gur RC, Gur RE, Davatzikos C (2007) COMPARE: Classification of Morphological Patterns Using Adaptive Regional Elements. IEEE Trans Med Imaging 26 93–105. 10.1109/TMI.2006.886812 17243588

[28] Liu F, Guo W, Yu D, Gao Q, Gao K, et al (2012) Classification of Different Therapeutic Responses of Major Depressive Disorder with Multivariate Pattern Analysis Method Based on Structural MR Scans. Plos One 7: e40968. 22815880 10.1371/journal.pone.0040968 PMC3398877

[29] Pereira F, Mitchell T, Botvinick M (2009) Machine learning classifiers and fMRI: A tutorial overview. NeuroImage 45: S199–209. 19070668 10.1016/j.neuroimage.2008.11.007 PMC2892746

[30] Zhu CZ, Zang YF, Cao QJ, Yan CG, He Y, et al (2008) Fisher discriminative analysis of resting-state brain function for attention-deficit/hyperactivity disorder. NeuroImage 40: 110–120. 18191584 10.1016/j.neuroimage.2007.11.029

[31] Shen H, Wang L, Liu Y, Hu D (2010) Discriminative analysis of resting-state functional connectivity patterns of schizophrenia using low dimensional embedding of fMRI. NeuroImage 49: 3110–3121. 19931396 10.1016/j.neuroimage.2009.11.011

[32] Pagani M, Salmaso D, Rodriguez G, Nardo D, Nobili F (2009) Principal component analysis in mild and moderate Alzheimer’s disease–A novel approach to clinical diagnosis. Psychiatry Research: Neuroimaging 173: 8–14. 19443186 10.1016/j.pscychresns.2008.07.016

[33] Jafri MJ, Pearlson GD, Calhoun VD (2007) A maximal-correlation approach using ICA for testing functional network connectivity applied to schizophrena. Biomedical Imaging: From Nano to Macro ISBI: 468–471.

[34] Wang Y, Wu Y (2010) Face recognition using Intrinsicfaces. Pattern Recognition 43: 3580–3590.

[35] Vapnik V (1995) The natures of statistical learning theory. New York: Springer-Verlag.

[36] APA (2000) Diagnostic and statistical manual of mental disorders. 4th edn. Washington. DC: American Psychiatric Press.

[37] Kay SR, Fiszbein A, Opler LA (1987) The positive and negative syndrome scale (PANSS) for schizophrenia. Schizophr Bull 13: 261–276. 3616518 10.1093/schbul/13.2.261

[38] Hamilton M (1960) A rating scale for depression. J Neurol Neurosurg Psychiatry 23: 56–62. 14399272 10.1136/jnnp.23.1.56 PMC495331

[39] Tzourio-Mazoyer N, Landeau B, Papathanassiou D, Crivello F, Etard O, et al (2002) Automated anatomical labeling of activations in SPM using a macroscopic anatomical parcellation of the MNI MRI single-subject brain. NeuroImage 15: 273–289. 11771995 10.1006/nimg.2001.0978

[40] Achard S, Salvador R, Whitcher B, Suckling J, Bullmore E (2006) A resilient, low-frequency, small-world human brain functional network with highly connected association cortical hubs. J Neurosci 26: 63–72. 16399673 10.1523/JNEUROSCI.3874-05.2006 PMC6674299

[41] Hsu C-W, Lin C-J (2002) A comparison of methods for multiclass support vector machines. IEEE Transactions on Neural Networks 13: 415–425. 18244442 10.1109/72.991427

[42] Raichle ME, Mintun MA (2006) Brain work and brain imaging. Annu Rev Neurosci 29: 449–476. 16776593 10.1146/annurev.neuro.29.051605.112819

[43] Honey GD, Pomarol-Clotet E, Corlett PR, Honey RAE, Mckenna PJ, et al (2005) Functional dysconnectivity in schizophrenia associated with attentional modulation of motor function. Brain 128: 2597–2611. 16183659 10.1093/brain/awh632 PMC3838931

[44] Mørch N, Hansen LK, Strother SC, Svarer C, Rottenberg DA, et al (1997) Nonlinear versus linear models in functional neuroimaging: learning curves and generalization crossover. Lect Notes Comput Sci 1230: 259–270.

[45] Micheloyannis S, Pachou E, Stam C (2006) Small-world networks and disturbed functional connectivity in schizophrenia. Schizophr Res 87: 60–66. 16875801 10.1016/j.schres.2006.06.028

[46] Friston KJ (1998) The disconnection hypothesis. Schizophr Res 30: 115–125. 9549774 10.1016/s0920-9964(97)00140-0

[47] Greicius MD, Krasnow B, Reiss AL, Menon V (2003) Functional connectivity in the resting brain: A network analysis of the default mode hypothesis. Proc Natl Acad Sci USA 100: 253–258. 12506194 10.1073/pnas.0135058100 PMC140943

[48] Raichle ME (2001) A default mode of brain function. Proc Natl Acad Sci USA 98: 676–682. 11209064 10.1073/pnas.98.2.676 PMC14647

[49] Botvinick MM, Braver TS, Barch DM, Carter CS, Cohen JD (2001) Conflict monitoring and cognitive control. Psychol Rev 108: 624–652. 11488380 10.1037/0033-295x.108.3.624

[50] Koshino H, Minamoto T, Ikeda T, Osaka M, Otsuka Y, et al (2011) Anterior medial prefrontal cortex exhibits activation during task preparation but deactivation during task execution. Plos One 6: e22909. 21829668 10.1371/journal.pone.0022909 PMC3148238

[51] Squire LR, Stark CE, Clark RE (2004) The medial temporal lobe. Annu Rev Neurosci 27: 279–306. 15217334 10.1146/annurev.neuro.27.070203.144130

[52] LaBar KS, Cabeza R (2006) Cognitive neuroscience of emotional memory. Nat Rev Neurosci 7: 54–64. 16371950 10.1038/nrn1825

[53] Talamini LM, Meeter M, Elvevag B, Murre JM, Goldberg TE (2005) Reduced parahippocampal connectivity produces schizophrenialike memory deficits in simulated neural circuits with reduced parahippocampal connectivity. Arch Gen Psychiatry 62: 485–493. 15867101 10.1001/archpsyc.62.5.485

[54] Tuula I, Tero T, Hasse K, Hannu L, Kirsi-Marja L, et al (1999) Diagnostic efficiency of the Rorschach schizophrenia and depression indices in identifying first-episode schizophrenia and severe depression. Psychiatry Res 87: 183–192. 10579551 10.1016/s0165-1781(99)00061-x

[55] Kohler C, Gur RC, Swanson CL (1998) Depression in schizophrenia: I. Association with neuropsychological deficits. Biol Psychiatry 43: 165–172. 9494697 10.1016/S0006-3223(97)00033-4

[56] Buchanan RW, Vladar K, Barta PE, Pearlson GD (1998) Structural evaluation of the prefrontal cortex in schizophrenia. Am J Psychiatry 155: 1049–1055. 9699693 10.1176/ajp.155.8.1049

[57] Buchsbaum MS, Hazlett EA (1998) Positron emission tomography studies of abnormal glucose metabolism in schizophrenia. Schizophr Bull 24: 343–364. 9718628 10.1093/oxfordjournals.schbul.a033331

[58] Bearden CE, Hoffman KM, Cannon TD (2001) The neuropsychology and neuroanatomy of bipolar affective disorder: a critical review. Bipolar Disord 3: 106–150. 11465675 10.1034/j.1399-5618.2001.030302.x

[59] Guo WB, Liu F, Xue ZM, Yu Y, Ma CQ, et al (2011) Abnormal neural activities in first-episode, treatment-naïve, short-illness-duration, and treatment-response patients with major depressive disorder: A resting-state fMRI study. J Affect Disord 135: 326–331. 21782246 10.1016/j.jad.2011.06.048

[60] Picard H, Amado I, MoucherMages S, Olié J-P, Krebs M-O (2008) The role of the cerebellum in schizophrenia: an update of clinical cognitive, and functional evidences. Schizophr Bull 34: 155–172. 17562694 10.1093/schbul/sbm049 PMC2632376

[61] Schmahmann JD, Caplan D (2006) Cognition, emotion and the cerebellum. Brain 129: 290–292. 16434422 10.1093/brain/awh729

[62] Andreasen NC, Rezai K, Alliger R, Swayze VI, Flaum M, et al (1992) Hypofrontality in neuroleptic-naive patients and in patients with chronic schizophrenia: Assessment with xenon 133 single photon emission computed tomography and the Tower of London. Arch Gen Psychiatry 49: 943–958. 1360199 10.1001/archpsyc.1992.01820120031006

[63] Menon V, Anagnoson RT, Mathalon DH, Glover GH, Pfefferbaum A (2001) Functional neuroanatomy of auditory working memory in schizophrenia: Relation to positive and negative symptoms. NeuroImage 13: 433–446. 11170809 10.1006/nimg.2000.0699

[64] Landro NI, Stiles TC, Sletvold H (2001) Neuropsychological function in nonpsychotic unipolar major depression. Neuropsychiatry Neuropsychol Behav Neurol 14: 233–240. 11725217

[65] Merriam E, Thase M, Haas G, Keshavan M, Sweeney JA (1999) Prefrontal cortical dysfunction in depression determined by Wisconsin card sorting test performance. Am J Psychiatry 156: 780–782. 10327916 10.1176/ajp.156.5.780

[66] Barch DM, Sheline YI, Csernansky JG, Snyder AZ (2003) Working Memory and Prefrontal Cortex Dysfunction: Specificity to Schizophrenia Compared with Major Depression. Society of Biological Psychiatry 53: 367–384. 10.1016/s0006-3223(02)01674-8 12614990

[67] Ding SL, Van HGW, Cassell MD, Poremba A (2009) Parcellation of human temporal polar cortex: a combined analysis of multiple cytoarchitectonic, chemoarchitectonic, and pathological markers. J Comp Neurol 18: 595–623. 10.1002/cne.22053 PMC3665344 19363802

[68] Phelps EA (2004) Human emotion and memory: interactions of the amygdala and hippocampal complex. Current Opinion in Neurobiology 14: 198–202. 15082325 10.1016/j.conb.2004.03.015

[69] Siever LJ (2008) Neurobiology of aggression and violence. Am J Psychiatry 165: 429–442. 18346997 10.1176/appi.ajp.2008.07111774 PMC4176893

[70] Wright IC, Rabe-Hesketh S, Woodruff PW, David AS, Murray RM, et al (2000) Metaanalysis of regional brain volumes in schizophrenia. Am I Psychiatry 157: 16–25. 10.1176/ajp.157.1.16 10618008

[71] Savitz J, Drevets WC (2009) Bipolar and major depressive disorder: neuroimaging the develop mental-degenerative divide. Neurosci Biobehav Rev 33: 699–771. 19428491 10.1016/j.neubiorev.2009.01.004 PMC2858318

[72] Hamazaki K, Hamazaki T, Inadera H (2012) Fatty acid composition in the postmortem amygdala of patients with schizophrenia, bipolar disorder, and major depressive disorder. Psychia Res 46: 1024–1028. 10.1016/j.jpsychires.2012.04.012 22572570

[73] Hinkley LBN, Vinogradov S, Guggisberg AG, Fisher M, Findlay AM, et al (2011) Clinical Symptoms and Alpha Band Resting-State Functional Connectivity Imaging in Patients With Schizophrenia: Implications for Novel Approaches to Treatment. Biol Psychiatry 70: 1134–1142. 21861988 10.1016/j.biopsych.2011.06.029 PMC3327723

[74] Fox MD, Zhang D, Snyder AZ, Raichle ME (2008) Global signal regression and anticorrelations in resting state fMRI data. Proc HBM 575 W-AM.

[75] Murphy K, Birn RM, Handwerker DA, Jones TB, Bandettini PA (2009) The impact of global signal regression on resting state correlations: Are anti-correlated networks introduced? NeuroImage 44: 893–905. 18976716 10.1016/j.neuroimage.2008.09.036 PMC2750906

